# On-Surface Observation of the Formation of Organometallic Complex in a Supramolecular Network

**DOI:** 10.1038/srep10972

**Published:** 2015-06-10

**Authors:** Yibao Li, Linxiu Cheng, Chunhua Liu, Wei Liu, Yulan Fan, Xiaolin Fan, Qingdao Zeng

**Affiliations:** 1 Key Laboratory of Organo-pharmaceutical Chemistry, Gannan Normal University, Ganzhou 341000, P. R. China; 2 CAS Key Laboratory of Standardization and Measurement for Nanotechnology, National Center for Nanoscience and Technology (NCNST), Beijing 100190, P. R. China; 3 Material and Chemical Engineering Department, Pingxiang University, Pingxiang 337055, China

## Abstract

The on-surface formation of organometallic monomers or oligomers, especially in supramolecular network, attracts an extensive interest for chemists and material scientist. In this work, we have investigated metal coordination between zinc (II) phthalocyanine (ZnPc) and 1, 3-di (4-pyridyl) propane (dipy-pra) in the 2, 6, 11-tricarboxydecyloxy-3, 7, 10-triundecyloxy triphenylene (asym-TTT) supramolecular template by means of scanning tunneling microscopy (STM) on highly oriented pyrolytic graphite (HOPG) substrate under ambient conditions. The experimental results demonstrate that every two ZnPc molecules in one nano-reactor connect with each other through one dipy-pra molecule by metal-coordination interaction. In this coordinating process, the template of asym-TTT supramolecular networks plays a significant role.

Recently, a new field of chemistry featuring weak interactions, coordination bonding, and covalent chemistry at solid surfaces has drawn much attention because of its capacity of generating two-dimensional nanostructures with novel functions providing potential applications^1^,^2^,^3^,^4^,^5^,^6^,^7^. In particular, the metal-organic coordination has been regarded as an effective way to design the materials, for this method offers the flexibility of using non-covalent interactions to promote the formation of well-defined molecular frameworks^8^,^9^,^10^,^11^. Meanwhile, modern surface science is revolutionized after the introduction of scanning tunneling microscopy (STM), which proves to be a useful tool for the direct observation of the nature of surface phenomena. Therefore, the combination of surface-confined chemistry and scanning tunneling microscopy (STM) techniques leads to the study of metal coordination at the nanometer level^12^,^13^.

Recently, many self-assemblies of metal-organic complex have been reported^14^,^15^,^16^,^17^, such as coordination properties of metalloporphyrins physisorbed on surfaces^18^,^19^,^20^, metallo-Pcs bridged by bipyridines^21^,^22^. In these years, Zinc (II) phthalocyanine (ZnPc) has received great attention due to its low degree of distortion from planarity, charming electro-optical, magnetic properties and the ability of binding affinity toward many different ligands containing N compounds^23^,^24^. As ligands, owing to the strong metal-coordination interaction between the metal atoms and nitrogen atom of pyridine^25^,^26^, pyridines (Pys) have become promising candidates of ligand for constructing molecular architectures. Previous studies focused mainly on the metal-coordination in open environments, that is, the ligand can be mobilized freely so as to form varieties of stable assemblies. These investigations proceeded in solution or on surface, not in cavities of supramolecualr networks, which are generated from self-assembly of organic molecules. In contrast, the molecular species in confined space can strongly influence the interaction pathways compared to homogenous and bulk conditions. Recently, Zeng and co-workers have reported the supramolecular coordination of ZnPc with bipyridine in a nano-reactor of TCDB H-bonded network through a two-step coordination process at liquid/solid interface^22^. The whole coordination process was regulated by the synergies of ligand and template.

In this paper, the template effect has been further investigated by using supramolecular networks of asym-TTT molecules^27^. With this purpose, we have observed the process of metal-coordination between ZnPc and 1, 3-di (4-pyridyl) propane (dipy-pra) (Fig. 1) in the nano-cavities of 2, 6, 11- tricarboxydecyloxy-3, 7, 10- triundecyloxy triphenylene (asym-TTT) networks through the STM technique at liquid/solid interface. Moreover, *in situ* STM observation is also performed to give an insight into the template effect during the coordination process.

## Results and Discussion

**Figure 2:**
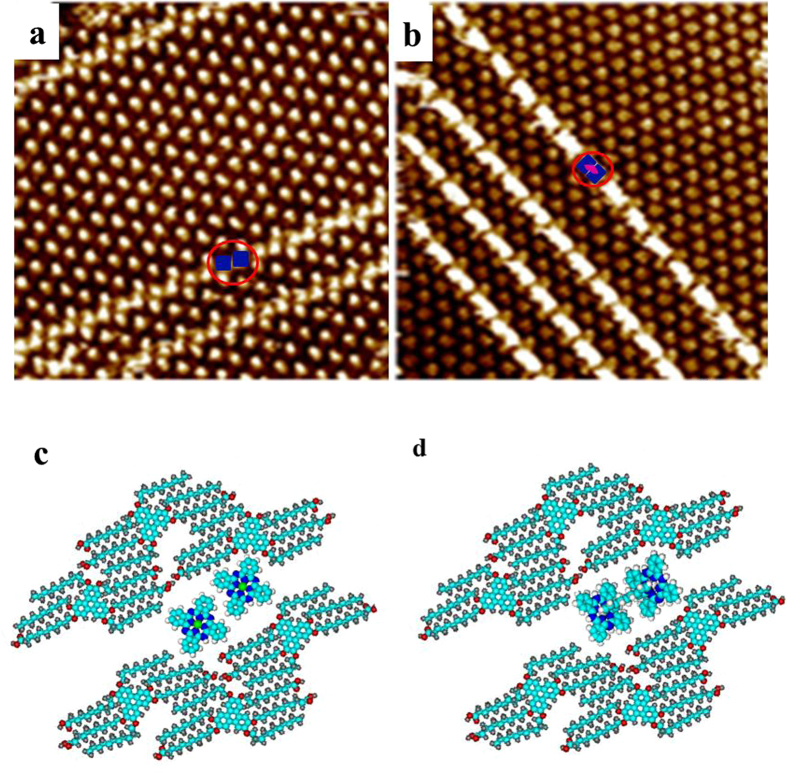
(**a**) STM image showing assembled structure of asym-TTT/Zn-Pc (42.2 nm × 42.2 nm, *I*_set_ = 198.4 pA, *V*_bias_ = 600.0 mV). (**b**) STM image showing entirely assembled structure of asym-TTT /Zn-Pc/dipy-pra system after the dipy-pra molecule was added (37.9 nm × 37.9 nm, *I*_set_ = 247.1 pA, *V*_bias_ = 1037 mV). (**c**) Molecular model for the observed area in (**a**). (**d**) Molecular model for the observed area in (**b**).

The self-assembled structure of asym-TTT/ZnPc has been previously reported^27^. Upon addition of ZnPc to the already formed asym-TTT supramolecular network at the solid/liquid interface, long molecular arrays of ZnPc are observed (Fig. 2a). The STM images as well as DFT calculations reveal the preferential adsorption of ZnPc dimers in the anisotropic rearrangement of an asym-TTT supramolecular network. As marked by the red circle in Fig. 2a, the blue rectangle can be ascribed to the ZnPc dimer, and the size of each ZnPc molecule is (1.2 ± 0.1) nm × (1.2 ± 0.1) nm.

Prior to investigating the template effect on metal-coordination, we firstly immobilize the ZnPc into the nano-cavities of supramolecular network. On this basis, a droplet (0.4 μL) of toluene solution containing dipy-pra (<10^−5^ M) is added. Then a new kind of long molecular arrays consisting of brighter rectangles has been observed (Fig. 2b). The brighter rectangles are connected into a whole, which is attributed the new organometallic complex (indicated by the red circle). The dimension of bright rectangles has measured to be ((3.0 ± 0.1) nm × (1.2 ± 0.1) nm). This axis of the organometallic complexes inside the cavity is tilted with the identical angle to the direction of 1D molecular arrays compared to the the ZnPc dimers. According to above analysis, it is deduced that this organometallic complex is formed by the ZnPc dimer, which is connected parallelly with one dipy-pra molecule through the zinc-pyridine coordination interactions.

**Figure 3:**
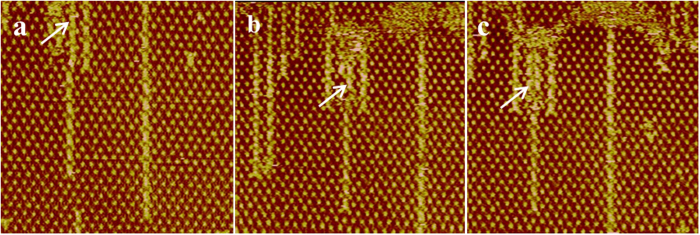
Sequential STM images of a 54.2 nm × 54.2 nm region illustrating the metal-coordination process of ZnPc in the rearranged networks. The time of the first image is arbitrarily set to zero (**a**), and the white arrows in the images (**a**–**c**) highlight the process.

To further investigate the coordination process of ZnPc/dipy-pra in molecular arrays, *in situ* STM experiments are performed at the same condition. Upon continued imaging the same region at different time after adding the dipy-pra molecules, the coordination process of ZnPc/dipy-pra can be clearly recorded (Fig. 3a–c). Along the white arrow in Fig. 3a, two ZnPc molecules are trapped in the cavity of the network. Although some coordinated ZnPc/dipy-pra molecular arrays can be observed beside the cavity, the coordination in the cavity which is marked by white arrow has not yet happened. In Fig. 3b, a large portion of ZnPc molecules has coordinated with dipy-pra molecules. It is clearly observed that a new kind of assembled structure marked by white arrow, where every two ZnPc molecules coordinated with one dipy-pra molecule, is generated in the cavity. In the last figure, we can see that all the ZnPc molecules have coordinated with dipy-pra molecules in cavities of the supramolecular asym-TTT networks (Fig. 3c). It is noted that the complex of ZnPc/dipy-pra generated in the adjacent cavity (indicated by white arrow) is identical to the newly formed complex mentioned in Fig. 3b. On this basis of above phenomenon, we can speculate that the template plays an important effect on the coordinating process, in which every two ZnPc molecules bridge with one dipy-pra molecule through the zinc-pyridine interactions in one nano-reactor.

**Figure 4:**
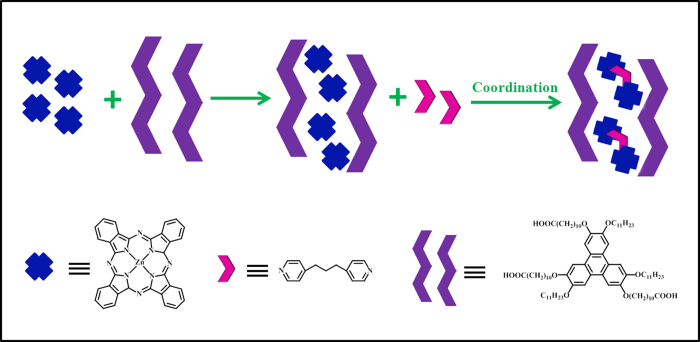
Schematic illustration of the formation of ZnPc/dipy-pra coordination.

According to the above STM results and analysis, a tentative model is proposed to illustrate the mechanism of the formation of ZnPc/dipy-pra complex (Fig. 4). Firstly, asym-TTT molecules can form two-dimensional well-defined nano-scaled networks, in which the molecules stand together to form antiparallel molecular lines with the assistance of hydrogen bonds provided by carboxyl at the terminal of asym-TTT molecules. Every two antiparallel molecular lines generate two zig-zag molecular chains, and then the adjacent molecular chains form condensed molecular grids with the action of van der Waals force between molecules. These network structures with controllable cavities are sensitive to guest molecules. Secondly, the introduction of ZnPc molecules breaks the van der Waals force between molecular chains. The parallel motions of molecular chains lead to the transformation of supramolecular network so as to be suitable to accommodate ZnPc dimers. As a result, the Zn-Pc dimers absorb in the newly formed cavities.

In the process of coordination, after the introduction of dipy-pra molecule, the cavity size remains constant, and one dipy-pra molecule tends to combine ZnPc molecules promptly. For one thing, the stability of the ZnPc dimer which is suitable for filling the cavity is higher than that of single ZnPc molecule in one cavity. In contrast, the cavity is too large for the single ZnPc molecule, which tends to flee from the cavity. For another, the dipy-pra molecule with two pyridines is apt to absorb two ZnPc molecules. As a result, ZnPc dimer with close distance between two ZnPc molecules has advantage in coordinating with dipy-pra molecule. Moreover, the size of the nano-reactor (cavity of asym-TTT networks) is relatively immobilized during the coordinating process, which confines the fluxion of ZnPc dimer and dipy-pra, and leads to the benefit of coordination.

In summary, we have studied metal-coordination between ZnPc and dipy-pra in the asym-TTT supramolecular nano-template. Upon following the asym-TTT/ZnPc/dipy-pra system on graphite surface, we *in situ* observe the coordination process that every two ZnPc molecules bridge with one dipy-pra molecule through the zinc-pyridine coordination interactions in one nano-reactor. During the coordinating process, the template of asym-TTT networks plays an important role. This result may provide some clues for recognizing surface reaction in nano-confined space.

## Methods

The asym-TTT was synthesized according to the reported procedures^27^. ZnPc, dipy-pra and 1-octanoic acid were purchased from TCI Company, and all these materials were used without any further purification. Asym-TTT, ZnPc, and dipy-pra were dissolved in toluene with concentration less than 10^−5^ M. Firstly, a droplet (0.4 μL) of the toluene solution containing asym-TTT was deposited onto a freshly cleaved surface (5 mm × 5 mm) of highly oriented pyrolytic graphite (HOPG, grade ZYB, Advanced Ceramics Inc., Cleveland, USA), and then a droplet (0.4 μL) of the 1-octanoic acid was added into the studied sample. A few minutes later, the STM investigation was performed. It can form well-ordered two-dimension self-assembly nanostructure. And then a droplet (0.4 μL) of the toluene solution containing ZnPc was deposited onto the studied sample. After 5 minutes later, the sample was studied by STM. Secondly, a droplet (0.4 μL) of toluene solution containing dipy-pra (<10^−5^ M) was added into the studied sample. After 20 minutes later, the STM investigation was performed.

The STM measurements were performed by using a Nanoscope IIIa (Bruker, Germany) under ambient conditions. All STM images presented were recorded in constant current mode using a mechanically cut Pt/Ir (80/20) tip. Toluene as solvent, the sample with concentration (1.0 × 10^−5^ M) was prepared for following experiment.

## Additional Information

**How to cite this article**: Li, Y. *et al.* On-Surface Observation of the Formation of Organometallic Complex in a Supramolecular Network. *Sci. Rep.* **5**, 10972; doi: 10.1038/srep10972 (2015).

## Figures and Tables

**Figure 1:**
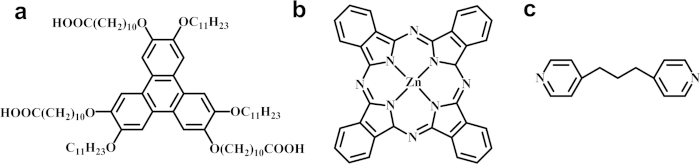
Chemical structures of (**a**) asym-TTT, (**b**) ZnPc and (**c**) dipy-pra.
